# The association between obesity and foot pain: metabolic, biomechanical or both?

**DOI:** 10.1186/1757-1146-8-S2-O5

**Published:** 2015-09-22

**Authors:** Paul A Butterworth, Hylton B Menz, Donna M Urquhart, Flavia M Cicuttini, Julie A Pasco, Sharon L Brennan, Anita E Wluka, Boyd J Strauss, Joseph Proietto, John B Dixon, Graeme Jones, Karl B Landorf

**Affiliations:** 1 Department of Podiatry, La Trobe University, Bundoora, Victoria, Australia; 2 Lower Extremity and Gait Studies Program, La Trobe University, Bundoora, Victoria, Australia; 3 School of Health and Human Sciences, Southern Cross University, Bilinga, Queensland, Australia; 4 Department of Epidemiology and Preventive Medicine, School of Public Health and Preventive Medicine, Monash University, Melbourne, Victoria, Australia; 5 School of Medicine, Deakin University, Geelong, Victoria, Australia; 6 North-West Academic Centre, University of Melbourne, Melbourne, Victoria, Australia; 7 Australian Institute of Musculoskeletal Sciences, Melbourne, Victoria, Australia; 8 University of Melbourne and Austin Health Melbourne, Melbourne, Victoria, Australia; 9 Baker IDI Heart and Diabetes Institute and Alfred Hospital, Melbourne, Victoria, Australia; 10 Menzies Research Institute, Hobart, Tasmania, Australia

**Keywords:** Foot, pain, obesity

## Background

Foot pain is a common complaint amongst adults. Foot pain has been associated with body and fat mass, as well as foot function. We conducted a series of studies to investigate the relationship between these variables and foot pain.

## Process

Initially, two systematic reviews were undertaken to assess: (i) the relationship between body mass index with musculoskeletal foot disorders, and (ii) the relationship between body composition and foot structure and function. Following this, we undertook a longitudinal and cross-sectional study of fat mass and foot pain to determine any association. Finally, a cross-sectional study of foot posture, range of motion and plantar pressure characteristics in obese and non-obese individuals was undertaken.

## Findings

The findings of this work demonstrate that in adults:

- General foot pain and plantar heel pain is strongly associated with increasing body mass index

- Obesity is strongly associated with planus (low-arched) foot posture, pronated dynamic foot function and increased plantar pressures when walking

- Obese individuals exhibited flatter feet, reduced inversion-eversion range of motion, and higher peak plantar pressures

- Body weight is independently associated with plantar loading after accounting for foot characteristics (e.g. under the midfoot)

- Fat mass, not fat-free mass, is a predictor of foot pain; thus, foot pain in overweight and obese individuals may be attributed to metabolic and biomechanical factors

## Conclusions

Increased fat mass is significantly associated with foot pain and increased body mass is associated with poor foot function. Considering that the prevalence of obesity is increasing worldwide, the incidence of musculoskeletal foot disorders is also likely to increase. Therefore, the role of the podiatrist should include appropriate discussions with patients and health practitioners regarding the association between obesity and foot pain.

